# Proceedings of the Society

**Published:** 1856-07

**Authors:** 


### QUARTERLY REPORT OF PROCEEDINGS.

### PAPERS READ AT THE ORDINARY MEETINGS.

*Monday, April 7th*, 1856.—" On Concurrent Scarlet Fever and Measles." By W. B. Kesteven, Esq.

The Report of the Epizootic Committee of the Epidemiological Society on Pleuropneumonia among Cattle.

*Monday, May 5th.*—" On the Geographical Distribution of Health and Disease in Connexion chiefly with Natural Phenomena." By Alexander Keith Johnston, Esq., F.R.S.E.

*Monday, June 2nd.*—" Suggestions for Observations on the Natural Influence of Cholera in the Lower Animals." By Walter Lauder Lindsay, M.D., Perth.

### PRESENTATIONS.

Besides several works on epidemic diseases, the Society has received a valuable Map illustrating the Geographical Distribution of Health and Disease, from A. Keith Johnston, Esq., F.R.S.E.; and a Chart of the great Epidemics which have passed over Europe between the years A.M. 1348 and A.D. 1850, from Dr. Henry Kennedy of Dublin.

### NEW OFFICERS OF THE SOCIETY.

At the Annual Meeting in April, the following gentlemen were elected office-bearers for the session 1856-57:

*President.*—Benjamin Guy Babington, M.D. F.R.S. *Vice-Presidents.*—Thomas Addison, M.D.; Richard Bright, M.D., F.R.S.; Sir B. C. Brodie, Bart., F.R.S.; Sir Wm. Burnett, K.C.B., K.C.H., F.R.S.; Sir C. M. Clarke, Bart., M.D., F.R.S.; Rev. Thomas Dale, M.A.; R. D. Grainger, Esq., F.R.S.; Sir Charles Hastings, M.D., D.C.L.; Sir John Liddell, C.B., M.D., F.R.S.; John Nussey, Esq.; John Propert, Esq.; John Simon, Esq., F.R.S.; Andrew Smith, M.D.; Thomas Southwood Smith, M.D.; Colonel Sykes, V.P.R.S.; Thomas Watson, M.D. *Treasurer.*—Thomas Addison, M.D. *Honorary Secretaries.*—J. O. McWilliam, M.D., F.R.S., R.N.; J. H. Tucker, Esq. *Foreign and Colonial Secretaries.*—Belgium—A. Sayer, M.D.; East Indies—James Bird, M.D., C. Finch, M.D.; Egypt and Syria—William Camps, M.D.; France—Waller Lewis, M.B., F.G.S.; Germany and Russia—Hermann Weber, M.D., W. E. Swaine, M.D.; Portugal and the Brazils—J. O. McWilliam, M.D., R.N., F.R.S.; Sweden, Norway, Denmark, and Iceland—R. Gordon Latham, M.D., F.R.S.; West Indies and North America—G. Milroy, M.D. *Other Members of Council*—C. L. Bradley, Esq.; A. Bryson, M.D, F.R.S., R.N.; Burford Carlill, M.D.; W. D. Chowne, M.D.; Robert Cross, M.D.; Geo. Glover, Esq.; Headlam Greenhow, M.D.; E. Headland, Esq.; T. Hunt, Esq.; C. F. J. Lord, Esq.; J. F. Marson, Esq.; W. E. Murphy, M.D.; B. W. Richardson, M.D; E. C. Seaton, M.D.; F. Sibson, M.D., F.R.S.; J. Snow, M.D.; F. O. Ward, Esq.; J. Waters, M.D.

